# The relationship between epigenetic age and the hallmarks of aging in human cells

**DOI:** 10.1038/s43587-022-00220-0

**Published:** 2022-05-16

**Authors:** Sylwia Kabacik, Donna Lowe, Leonie Fransen, Martin Leonard, Siew-Lan Ang, Christopher Whiteman, Sarah Corsi, Howard Cohen, Sarah Felton, Radhika Bali, Steve Horvath, Ken Raj

**Affiliations:** 1 Radiation Effects Department, Centre for Radiation, Chemical and Environmental Hazards, Public Health England, Chilton, Didcot, UK.; 2 Toxicology Department, Centre for Radiation, Chemical and Environmental Hazards, Public Health England, Chilton, Didcot, UK.; 3 The Francis Crick Institute, London, UK.; 4 Elizabeth House Surgery, Warlingham, Surrey, UK.; 5 Department of Dermatology, Churchill Hospital, Oxford, UK.; 6 Department of Human Genetics, David Geffen School of Medicine, University of California, Los Angeles, Los Angeles, CA, USA.; 7 Department of Biostatistics, Fielding School of Public Health, University of California, Los Angeles, Los Angeles, CA, USA.; 8 Altos Labs, Cambridge Institute of Science, Cambridge, UK.; 9 Present address: Altos Labs, Cambridge Institute of Science, Cambridge, UK.; 10 These authors contributed equally: Sylwia Kabacik, Donna Lowe.; 11 These authors jointly supervised this work: Steve Horvath, Ken Raj.

## Abstract

Epigenetic clocks are mathematically derived age estimators that are based on combinations of methylation values that change with age at specific CpGs in the genome. These clocks are widely used to measure the age of tissues and cells^1,2^. The discrepancy between epigenetic age (EpiAge), as estimated by these clocks, and chronological age is referred to as EpiAge acceleration. Epidemiological studies have linked EpiAge acceleration to a wide variety of pathologies, health states, lifestyle, mental state and environmental factors^2^, indicating that epigenetic clocks tap into critical biological processes that are involved in aging. Despite the importance of this inference, the mechanisms underpinning these clocks remained largely uncharacterized and unelucidated. Here, using primary human cells, we set out to investigate whether epigenetic aging is the manifestation of one or more of the aging hallmarks previously identified^3^. We show that although epigenetic aging is distinct from cellular senescence, telomere attrition and genomic instability, it is associated with nutrient sensing, mitochondrial activity and stem cell composition.

The first multitissue clock developed for in vivo use was based on DNA methylation profiles derived from over 50 human tissues^1^. Although it can be used to estimate EpiAge of cultured cells, the projected EpiAge was slightly skewed from chronological age. To address this problem, a second multitissue clock (Skin&blood clock) that can just as accurately estimate age of cells in vivo as well as in vitro was generated^4^. It outperforms other epigenetic clocks in in vitro experiments in terms of accuracy and reliability (Extended Data Fig. 1). Therefore, age analyses carried out with the Skin&blood clock will be shown here, whereas those by Horvath, Hannum and PhenoAge clocks are included as extended data figures for the purpose of comparison and not as bases of conclusions. The Skin&blood clock’s performance was previously demonstrated to be highly accurate across different tissues, including the skin^4^, be it full thickness, epidermis (keratinocytes) or dermis (fibroblasts) (Fig. 1a). Importantly, basal cell carcinomas (BCCs) exhibited largely younger EpiAge than corresponding healthy tissue from the same donor (Fig. 1b), indicating that as with the Horvath clock, the Skin&blood clock connects with some underlying biological mechanisms that are linked with age and become disrupted in disease state such as BCC and progeria^4^. Importantly, this clock also accurately measures age of cells in vitro and tracks their aging in culture (Fig. 1c), permitting its use in the in vitro experiments described below to elucidate the mechanisms of aging.

The link between cellular senescence and aging is indisputable. Several processes, including telomere attrition^5,6^, unrepaired DNA damage^7^ and overexpression of oncogenes^8^, can induce senescence. We investigated whether EpiAge is associated with these. Primary human dermal fibroblasts (HDFs) from 14 healthy neonatal donors, were each partitioned into four populations: control, X-irradiated (20 Gy), Ras-transduced and those destined for replicative senescence. Radiation or oncogene overexpression induced all donor cells to senesce with EpiAge close to zero, as were the controls (Fig. 1d and Extended Data Fig. 2). However, cells that underwent replicative senescence exhibited increased EpiAge. Importantly, these cells were cultured for up to 6 months before they became senescent, whereas irradiated and Ras-transduced cells became senescent and were collected for analysis within 2 weeks. This difference in culture time is evident from the DNA methylation-based estimation of telomere length^9^ of these cells (Fig. 1e). The telomeres of replicative senescent cells were considerably shorter than those of the other senescent and control cells collected at the start of the experiment. Therefore, the increased EpiAge of replicative senescent cells could be due to aging during their time in culture, as shown to occur in Fig. 1c, rather than to their state of senescence. To test this, we transduced HDFs and human coronary artery endothelial cells (HCAECs) with the catalytic subunit of the telomerase enzyme, hTERT. As expected, these cells proliferated without undergoing replicative senescence even after over 60 cumulative population doublings (CPD), whereas their counterparts transduced with empty vector eventually stopped proliferation and underwent replicative senescence. The EpiAge of both hTERT-transduced and control cells increased with time in culture (Fig. 1f,g and Extended Data Fig. 3), and the hTERT-transduced cells continued to age up until the last time point when the experiment was halted, proving that the increased EpiAge of replicative senescent cells is owed not to the state of senescence but to the aging of the cells during their culture. Importantly, hTERT, which prevents telomere attrition, does not prevent or impede the rate of epigenetic aging. Collectively, these experiments demonstrate that epigenetic aging, as measured by the Skin&blood clock, is distinct from two of the best-characterized hallmarks of aging, cellular senescence and telomere attrition.

Another feature thought to be an important contributor of aging is genomic instability^3^, which can be instigated by different means, including ionizing irradiation. Acute irradiation (20 Gy) of HDFs derived from 25 healthy neonatal donors did not induce any significant change to the EpiAge of the cells after 30 days (Fig. 2a). We tested lower doses of radiation delivered continuously to primary HDKs and HDFs. We previously determined the range of dose rates of ƴ-irradiation that is sufficient to inflict a small amount of double-strand DNA breaks on cells but does not prevent them from proliferating and growing^10^. At continuous ƴ-irradiation of 1 mGy/h, the growth of HDKs (Fig. 2b) and HDFs (Fig. 2d) was modestly impeded, but no significant alterations to their EpiAge were observed after continuous exposure to radiation for 70 and 150 days, respectively (Fig. 2c,e and Extended Data Fig. 4). This was also the case when cells were continuously irradiated at 20 mGy/h for three passages within 21 days (Fig. 2f). Next, we tested a pulsed DNA damage regimen, which was proposed to accelerate the aging of mouse embryonic fibroblasts (MEFs)^11^. Therefore, we exposed three different isolates of MEFs to 24 h of 50 mGy/h ƴ-irradiation followed by no radiation for a week. This method also did not induce any significant and consistent change to the EpiAge of the cells (Fig. 2g). These results, obtained through investigation using different primary human and mouse cells and multiple radiation doses and regimens, demonstrate that epigenetic aging, as measured by Skin&blood clock, is not affected by genomic instability induced by radiation-induced DNA breaks.

Another well-characterized hallmark of aging is perturbation of nutrient sensing. Caloric restriction of numerous animal species extends their lifespans and presumably slows down their rate of aging^12,13^. The mammalian target of rapamycin (mTOR) complex is one of the pivotal nodes in the underlying pathway^14^. Indeed, administration of rapamycin, which blocks mTOR activity, extends the lifespan of mice^15^. We previously demonstrated that rapamycin treatment retards the rate of epigenetic aging of neonatal HDKs^16^. Here, we subjected hTERT-immortalized human umbilical vein endothelial cells (HUVECs), which allow cells at very late time points from their isolation (hence greater EpiAge) to be challenged with rapamycin. Indeed, perturbing the nutrient-sensing pathway even at late time points in culture retarded the rate of epigenetic aging (Fig. 2h and Extended Data Fig. 5), confirming the connection of this aging hallmark to epigenetic aging.

The role of mitochondria in aging has been shown to be crucial^17,18^. To ascertain whether mitochondria impairment impacts epigenetic aging, we treated HDKs with carbonyl cyanide m-chlorophenylhydrazone (CCCP), which reduces mitochondrial activity^19^. When JC-1 monomers were administered to control cells, they aggregated and fluoresced red in the mitochondria because of the high membrane potential of these organelles. In CCCP-treated cells however, monomeric JC-1 remained primarily green due to low aggregation in mitochondria because of reduced membrane potential (Fig. 3a). The impairment of mitochondrial function in CCCP-treated cells was confirmed empirically (Fig. 3b), and these cells exhibited highly accelerated epigenetic aging (Fig. 3c). Consistently, treatments of cells with Bezafibrate, which increases mitochondrial biogenesis^20^, slowed down the rate of epigenetic aging (Fig. 3d and Extended Data Fig. 6) and extended the proliferative capacity of HDKs (Fig. 3e). Collectively, these results indicate that epigenetic aging, in addition to being related to nutrient sensing, is also associated with mitochondrial activity, raising the intriguing possibility of a link between these two hallmarks of aging.

As cellular reservoirs of tissues, stem cell involvement in aging is intuitive. To interrogate the relationship between stem cells and epigenetic aging, we enriched HDK stem cells from neonatal skins of two donors. The stem cell-enriched fraction expressed significantly greater amounts of stem cell markers, ITGA6 (ref. ^21^), COL17A1 (ref. ^22^) and p63 (ref. ^23^) (Fig. 3f) and was younger than the stem cell-depleted fraction (Fig. 3g and Extended Data Fig. 7). This age difference was further increased when these cell fractions isolated from another four independent donors were put to culture in vitro until confluence (Fig. 3h and Extended Data Fig. 7). The consistency of these results makes a strong case that EpiAge of a tissue is a result of the combined methylation profiles of its constituent cells, with stem cells exhibiting younger age than non-stem cells. This echoes a similar observation with mouse muscle stem cells^24^ and human intestinal stem cells^25^.

If EpiAge of a tissue is a composite of different ages of its cells, then it would follow that considerably younger and older cells may be present within the same tissue. To investigate this idea, we generated cell clones from HCAECs derived from two different donors with an EpiAge of 23 years. Clones derived from single cells were isolated and expanded to confluence. The EpiAges of these clones were considerably different, flanking both sides of the EpiAge (23 years) of the parental cell populations (Fig. 3i), and the observed EpiAge heterogeneity was much greater than the variability of the Skin&blood clock (Fig. 3j). That younger and older cell populations can be derived from a single-cell population underlines the crucial contribution of cell composition to the measurement of EpiAge.

The above observation indicates what constitutes EpiAge, but not when the epigenetic clock starts ticking. When the first multi-tissue epigenetic clock was reported, its application on embryonic stem cells (ESCs) and induced pluripotent stem cells (iPSCs)^26^ in culture showed that despite extensive proliferation, these cells do not undergo epigenetic aging^1^. Therefore, they are natural starting points in the search for when the ticking initiates. We differentiated human ESCs into endothelial cells (Fig. 4a) and tracked their EpiAge in culture. It is evident that the epigenetic clock starts ticking very early on from the point of differentiation (Fig. 4b and Extended Data Fig. 8a–d). Interestingly, this was accompanied by a clear and progressive reduction in genome-wide mean methylation levels (Fig. 4c), indicating substantial change to the epigenetic landscape of the cells, triggered by the differentiation-inducing factors. Similarly, differentiation of ESCs into neural progenitor cells also triggered the initiation of the clock (Fig. 4d and Extended Data Fig. 8e–h). To obtain a greater resolution, we induced iPSC differentiation in lung epithelial cells. This differentiation pathway is better characterized, with intermediate states of anterior foregut endoderm (AFE) cells and lung progenitor (LP) cells before the attainment of basal epithelial cells. Time-course age analyses show once again that the epigenetic clock starts ticking very soon after differentiation, when cells cease expression of Oct4 and initiate expression of p63 (Fig. 4e and Extended Data Fig. 9). As such, epigenetic aging is a part of the process of life that begins from the early moments of ESC differentiation. This finding is supported by reports that differentiation of retinal cells in vitro is accompanied by an increase in EpiAge^27^. The trajectory of epigenetic aging during embryogenesis may not be linear, as there is reportedly a short phase of rejuvenation followed by aging^28^.

The observation that aging begins so early in life is possible because age can now be measured based on the biology of the cell instead of the passing of time. This measurement allows interrogation of the link between age and longevity. Here, we observed that although it is true that perturbations such as rapamycin treatment, which reduces the rate of aging, also extends the proliferative capacity^16^, hence the lifespan of the cell population, this is not so under all circumstances. Specifically, nicotinamide adenine dinucleotide (NAD), nicotinamide riboside (NR) and metformin can all extend the lifespan of cellular populations (Fig. 4f and Extended Data Fig. 10) but do not significantly alter the rate of epigenetic aging. Consolidating these results (Fig. 4g) with previously reported ones^16^, it appears that within the limited number of tested perturbations, those that affect the rate of epigenetic aging, as measured by the Skin&blood clock, also affect lifespan, but those that affect lifespan may not necessarily affect aging, indicating that aging and longevity, although intimately associated, may nevertheless be distinct, as previously suggested^29^. This idea is consistent with our findings that age-related CpGs and lifespan-related CpGs of mammals are almost entirely mutually exclusive^30^.

The excitement following the development of epigenetic clocks has been tinged with uncertainty as to the meaning of their measurements (i.e., EpiAge). This uncertainty is compounded by the fact that different epigenetic clocks appear to measure different features of aging^31^. Our investigations using the Skin&blood clock uncovered many features of epigenetic aging, of which two are particularly important. First, epigenetic aging initiates at very early point of life when pluripotency ceases. This process evidently continues through development, postnatal growth, maturity and adulthood until death, as epigenetic clocks are applicable to the entire lifespan. Therefore, epigenetic aging is not an auxiliary phenomenon but an integral part of the deterministic process of life. Despite this fact, epigenetic aging is not refractive to the influence of external factors that can alter its rate. Indeed, some experiments above demonstrate the malleability of the rate of epigenetic aging. At a higher level of consideration, the innate nature and inevitability of epigenetic aging contrasts with the stochasticity of wear and tear, which is presumed to exert a measurable aging effect only later in life when damage outstrips repair. This, however, does not argue against the relevance of wear and tear and cellular senescence. Instead, these distinct stochastic processes are likely to synergize with epigenetic aging in manifesting the overall phenotypical features of aging. If a successful strategy against aging is to be found, then these distinct and parallel aging mechanisms must be addressed; for example, by the removal of senescent cells^32^, together with the retardation of epigenetic aging.

The second pivotal point concerns the ticking of the clock. It is intuitive to assume that this ticking is owed to dynamic changes of methylation on age-related CpGs in all cells in a tissue. Our observations with cell clones suggest that the ticking of the epigenetic clock is, at the very least, a measure of change in cell composition with age. This change can perceivably occur through expansion or reduction of a subpopulation of cells with different ages within the tissue. It was previously shown that mouse muscle stem cells are considerably younger^24^. Therefore, such changes can conceivably result from alterations in the relative amounts of stem cells and non-stem cells, although the impact of stem cells from many more different tissues to aging requires further empirical investigations. There remains the possibility that the different ages of clones might be a result of epigenetic drift, which is not readily testable, although the similarity of age spread between clones from two independent experiments and donors would suggest a lower likelihood of this scenario, but not rule it out.

It was particularly important to address the question of the relationship between epigenetic aging and cellular senescence, as previous reports were equivocal in their conclusions^31,33,34^. Here, using primary cells from many individual donors, the results are clear that cellular senescence, although undoubtedly a major contributor to the aging phenotype, is not associated with epigenetic aging, as measured by the Skin&blood clock. Similarly, DNA damage and genomic instability have been hypothesized and proffered as means by which cells undergo epigenetic aging. Here, using different primary cell types derived from multiple donors, irradiated in different ways (acute or continuous) at different doses and dose rates, we did not observe any measurable impact on the rate of epigenetic aging.

The connection between epigenetic aging and nutrient sensing through mTOR is significant from several perspectives, particularly its agreement with a previous epigenome-wide associated study that identified SNPs within MLST8, a subunit of mTOR, as the locus that is most associated with epigenetic aging of the cerebellum^35^. The convergence of this epigenome-wide associated study approach with multiple cell culture experiments showing rapamycin-induced retardation of epigenetic aging greatly consolidates this association^16^ and also shows that epigenetic aging can be modulated, even at the older age spectrum. Corroborating this finding is the observation that caloric restriction retards epigenetic aging^36^, and different dietary constituents reportedly impact the EpiAge of mice^37^. Equally important is the influence of mitochondria function on epigenetic aging. Although this influence was indicated by experiments with immortalized cell lines^31^, the results here with primary human cells provide assurance of the involvement of mitochondria. The multifaceted activities of mitochondria necessitate more in-depth investigations to identify the precise routes by which this link is formed.

The two other hallmarks of aging not addressed here with the in vitro human system are loss of proteostasis and cell–cell communication. The connection of the latter with epigenetic aging was demonstrated in experiments with mice, where infusion of the plasma fraction from young rats into old rats rejuvenated the EpiAge of the latter^38^. This finding is further exemplified by the reduced rate of epigenetic aging in mice for which growth hormone receptor genes were ablated^39^.

Collectively, the results described here with primary cells from a large number of donors and multiple cell types, as well as in vivo mouse experiments previously reported^38,39^, indicate that nutrient sensing, mitochondrial function, stem cell exhaustion and altered cell–cell communication affect epigenetic aging as measured by Skin&blood clock, but cellular senescence, telomere attrition and genomic instability do not (Fig. 4h). The connection of epigenetic aging to four of the hallmarks of aging implies that these hallmarks are also mutually connected at a deeper level. If so, epigenetic clocks will be instrumental in identifying the underlying unifying mechanisms. The absence of a connection between the other aging hallmarks and epigenetic aging suggests that aging is a consequence of multiparallel mechanisms, crudely divided into deterministic pathways: those associated with epigenetic aging and stochastic ones, which are independent of epigenetic aging and may result instead from wear and tear. This brings together two long-argued causes of aging. It is clear that epigenetic clocks have and will continue to bring even greater clarity to the overall process of aging. The described work is limited by the absence of animal experiments, which are now made possible with the recent availability of mouse and universal mammalian epigenetic clocks.

## Methods

All experiments conducted were within the approval of the institutional ethics committee. In particular, human skin samples were obtained with ethical approval by the Oxford B Research Ethics Committee (reference 10/H0605/1), and informed and signed consent was obtained from the legal representatives of the donors before collection of tissues from neonatal foreskins derived from routine circumcision and healthy adult facial skin derived from minor dermatology procedures. No compensation was paid to the donors.

### Isolation and culture of primary human keratinocytes and fibroblasts.

Primary cells were isolated from human neonatal foreskin removed for routine circumcision or adult skin samples removed during dermatological procedures with ethical approval (Research Ethics Committee reference 10/H0605/1 IRAS 33408). Tissue were collected following a written consent by their legal representatives and was transported the same day and digested overnight at 4 °C with 0.5 mg ml^−1^ liberase DH (Roche, 5401089001) in CnT-07 Epithelial Proliferation Medium (CELLnTEC, CnT-07). The following day, the epidermis was removed using sterile instruments and then pressed in trypsin-EDTA to form a single-cell suspension, pelleted and resuspended in keratinocyte medium (CnT-07). Cells were seeded on collagen/fibronectin-coated flasks for keratinocyte isolation. To isolate fibroblasts, epidermis-free dermal pieces were grown in Dulbecco’s modified Eagle medium (DMEM) (Sigma-Aldrich, D6429) supplemented with 10% fetal bovine serum (FBS) (Gibco, 10500064) and grown as explants. Transport and initial isolation were carried out with double concentration of antibiotics, followed by 7 days in normal concentration (100 U penicillin, 0.1 mg streptomycin, 10 µg gentamycin and 0.25 μg amphotericin B per milliliter); routine culture and experiments were carried out without antibiotics. Cells were passed by washing with Hank’s balanced salt solution (HBSS) and trypsinization, followed by soybean trypsin inhibitor and resuspension in the appropriate medium and seeded at 1:3–1:5 ratio with coating used for initial isolation.

### Culture of endothelial cells.

HCAECs and HUVECs were purchased from Cell Applications and cultured in Meso Endo Cell Growth media (212–500) and Endothelial Cell media (211–500), respectively. These cells were cultured in sterile vessels precoated with 0.001% fibronectin (Sigma-Aldrich, F0895) in HBSS (Sigma-Aldrich, H6648) for at least an hour. For passaging, the cells were collected using Trypsin-EDTA (Sigma-Aldrich, T4174) and neutralized with soybean trypsin inhibitor (Invitrogen, 17075–029).

### Culture of MEFs.

Three different lots of MEF cells were purchased from Merck and ATCC. Cells were cultured and chronically irradiated in DMEM containing 20% FBS and 0.1 mM β-mercaptoethanol at 37 °C, 5% CO_2_ and 3% O_2_ conditions.

### In vitro epigenetic aging assay.

Cells (keratinocytes, fibroblasts and endothelial cells) were cultured in their respective media and precoated plates as described above. These cells were cultured in 10-cm dishes. When confluence was attained, they were collected with trypsin-EDTA and neutralized with soybean trypsin inhibitor (as above). Cell counts were obtained and recorded. Next, 200,000 (keratinocytes and fibroblasts) or 500,000 (endothelial cells) cells were seeded into fresh dishes. Remaining cells were either frozen in 10% dimethylsulfoxide (Sigma-Aldrich, D2438) in 90% FBS or pelleted and subjected to DNA extraction. Population doubling was ascertain by the formula; 3.32[log_10_(number of cells collected) − log_10_ (number of cells seeded)]. CPD is the sum total of all preceding population doublings. Compounds being tested (12.5 nM rapamycin, 2.5 nM CCCP, 75 µM bezafibrate, 20 µg ml^−1^ NAD, 10 µM NR and 25 µM metformin) were added to the media at each media change, which was carried out every other day. Assays were terminated when the control (untreated cells) ceased proliferating after 14 days in culture with repeated media change, indicating that the population of cells have entered the senescent state. DNA from the various passages of cells were extracted using the Zymo Quick DNA miniprep Plus kit (D4069). DNA was sent for profiling by Illumina EPIC array. The results (beta values) were analyzed and EpiAge estimated by the Skin&blood clock algorithm.

### Differentiation of ESCs into endothelial cells.

hESC line ESI-049 was purchased from ESI-BIO. Cells were cultured in a feeder-free conditions in StemFlex medium (Gibco, A3349401) in 6-well plates precoated with Geltrex (Gibco, A1413301) according to the manufacturer’s instructions. When colonies started to touch each other, cells were passed in clumps at ratio 1:20 using ReLeSR dissociation reagent (Stem Cell Technologies, 05872) according to the manufacturer’s instructions. For differentiation experiments, cells that were ready for passing were used. On day 1, Stem Flex medium was removed, cells were washed ones with HBSS and 3 ml N2B27 media (1:1 Neurobasal medium (Gibco, 21103049) and DMEM/F12 medium (Gibco,11330032), 0.5× N2 supplement (Gibco, 17502–048), 0.5× B27 supplement (Gibco, 17504–044), 2 mM L-glutamine (Gibco, 25030149) and 0.1 mM ß-mercaptoethanol (Gibco, 31350010), supplemented with 8 µM CHIR99021 (Sigma-Aldrich, SML1046–5MG) and 25 ng ml^−1^ BMP4 (ThermoFisher, PHC9534), was added. On day 4, medium was changed to StemPro-34 media (Gibco, 10639011) supplemented with 2 mM L-glutamine, 200 ng ml^−1^ VEGF (Peprotech, 100–20) and 2 µM Forskolin (Sigma-Aldrich, F3917–10MG). On day 6, medium was changed again for StemPro-34 media supplemented with 2 mM L-glutamine and 50 ng ml^−1^ VEGF. On day 8, cells were trypsinized and endothelial cells were purified using Dynabeads CD31 Endothelial Cell kit (Invitrogen, 11155D) according to the manufacturer’s instructions and seeded into a fibronectin-coated T25 flask in Meso Endo Cell Growth media.

### Differentiation of ESCs into neuronal progenitors.

H9 hESCs (Wicell Research Institute) were grown to 95% to 100% confluency and neuralized for a total of 12 days with a 1:1 mixture of N2- and B27-supplemented DMEM media (N2B27), to which 10 μM SB431542 and 100 nM LDN193189 (Sigma-Aldrich) were added. Cells were dissociated with Accutase (Life Technologies, A11105–01) and replated on day 7 in the same media plus 10 µM ROCK inhibitor (ROCKi; Sigma-Aldrich, Y0503) and on days 13, 16, 20 and 23 in N2B27 media plus 10 µM ROCKi. From day 20 onward, many forebrain progenitors expressing PAX6 (a cortical progenitor marker) and the proliferation marker MKI67 were observed in the cultures, whereas a small proportion of cells expressed neuronal differentiation markers such as ELAV4 and TUBB3. On day 23, cells were plated into poly-l-ornithine– coated (100 μg ml^−1^; Sigma-Aldrich, P6407) and laminin–coated plates (10 μg ml^−1^; Sigma-Aldrich, L2020) in B27 media plus 10 µM ROCKi supplemented with 10 μM Notch inhibitor, DAPT (*N*-[*N*-(3,5-difluorophenacetyl)-l-alanyl]-S-phenylglycine t-butyl ester), to induce cell cycle exit. On days 25 and 26, half of the media was replaced with half the initial volume of B27 plus 10 μM DAPT. From days 29 to 30 onward, half of the media was replaced with B27 every 5–6 days.

### Differentiation of iPSCs into lung epithelial cells.

iPSCs (StemBanc, SBAD3 and SBAD2) were obtained from NewCells Biotech under conditions of use outlined in the in3 MSCA-ITN project license agreement. iPSCs were maintained on Geltrex (ThermoFisher, A1413302)-coated 6-well plates in mTeSR1 medium (StemCell, 85850) prepared according to manufacturer’s instructions and passaged using EDTA-Versene (ThermoFisher, 15040066). On day 0, iPSCs were passaged to Geltrex-coated 12-well plates (split ratio 1:1 based on surface area) and differentiated to AFE cells using DMEM (ThermoFisher, 41965039)/MCDB-201 (Sigma-Aldrich, M6770) (1:1) medium supplemented with 0.25× LA-bovine serum albumin (Sigma-Aldrich, L9530), 0.25× ITS (Sigma-Aldrich, I3146), 100 U ml^−1^ Pen/Strep (ThermoFisher, catalog no. 15140122), 100 nM l-ascorbic acid (Sigma-Aldrich, A4544), 1 µM dexamethasone (Sigma-Aldrich, D4902) and 50 µM 2-mercaptoethanol (ThermoFisher, 31350010) and growth factors (Table 1). Cells were washed with PBS (Gibco, 14190–144) before medium was refreshed daily.

Between day 6 and day 15, DMEM/F12 (ThermoFisher, 31330) medium, supplemented with 100 U ml^−1^ with Pen/Strep, 50 µg ml^−1^ l-ascorbic acid, 0.05% bovine serum albumin (ThermoFisher, 15260–037), 1% Glutamax (ThermoFisher, 35050–061), 0.5% N2 (ThermoFisher, 17502–048), 1% B27 (ThermoFisher, 17504–044), 0.04 µg ml^−1^ 1-thioglycerol (Sigma-Aldrich, M6145), 3 µM CHIR99021 (Torcis, 4423), 10 ng ml^−1^ KGF (R&D Systems, 251-K), 10 ng ml^−1^ FGF10 (R&D Systems, 345-FG), 10 ng ml^−1^ BMP4 (R&D Systems, 314-BP), 20 ng ml^−1^ EGF (R&D Systems, 2028-EG) and 50 nM retinoic acid (Sigma-Aldrich, R2625) was refreshed every other day to differentiate AFE cells to LP cells.

On day 15, LP cells were digested with 0.05% trypsin (Sigma-Aldrich, T3924) for 1 min and resuspended in medium. Cloudy supernatant was removed and clumps were resuspended in DMEM/F12 medium supplemented with 100 U ml^−1^ Pen/Strep, 50 µg ml^−1^ l-ascorbic acid, 0.05% bovine serum albumin, 1% Glutamax, 0.5% N2, 1% B27, 0.04 µg ml^−1^ 1-thioglycerol, 3 µM CHIR99021, 10 ng ml^−1^ KGF and 10 ng ml^−1^ FGF10 and passaged to Matrigel-precoated 12-well plates at a 1:3 ratio. This medium was refreshed every other day until day 25.

On day 25, cells were passaged as single cells using 0.05% trypsin, Collagen IV (Sigma-Aldrich, catalog no. C6745)-precoated 12-well plates and KSFM (ThermoFisher, 17005042) medium, supplemented with 100 U ml^−1^ Pen/Strep, 0.5 ng ml^−1^ EGF, 25 µg ml^−1^ human albumin (Sigma-Aldrich, A9731), 1 µM A83–01 (Sigma-Aldrich, SML0788), 5 µM Y-27632 (Abcam, Ab120–129), 50 nM BMS 453 (Sigma-Aldrich, SML1149), 30 µg ml^−1^ BPE and 3 µM isoproterenol (Sigma-Aldrich, I5627). Basal-like cells could be maintained as single cells for at least 40 days or frozen using 9:1 FBS/dimethylsulfoxide.

DNA and RNA were isolated at different stages of differentiation using an AllPrep DNA/RNA Mini kit (Qiagen, 80204) in accordance with the manufacturer’s instructions.

### Gene expression analysis.

RNA was reverse transcribed to cDNA was using a random hexamer-based protocol and Maxima Reverse Transcriptase (ThermoFisher, catalog no. EP0742) in accordance with manufacturer’s instructions and gene expression was analyzed using SYBR green based real-time quantitative PCR (qRT-PCR) on the Quantstudio 6 Flex Real-Time PCR System (Applied Biosystems). The expression levels for Oct4 and p63 were calculated using ddCt method using GAPDH as a reference gene. Primers used in the experiment are listed in Table 2.

### Enrichment of keratinocyte stem cells.

Epidermis from neonatal foreskin samples prepared as described above was mechanically disaggregated in trypsin and incubated for 10 min at 37 °C to obtain a single-cell suspension. The cell suspension was passed through a 70-µm cell strainer to remove any clumps, and cells were counted. For estimation of EpiAge of cells isolated directly for epidermis, cells were resuspended in 6 ml CnT medium, added to 10-cm plate coated with 100 μg ml^−1^ collagen IV (Sigma-Aldrich, cat. C7521–5MG) and incubated for exactly 20 min at room temperature. After incubation, unattached cells were collected and labeled as stem cell-depleted fraction. The cells which attached to the plate were washed extensively, and the remaining fraction was trypsinized and collected as stem cell-enriched fraction; both fractions were used for DNA extraction and EpiAge measurement. To estimate the EpiAge of the stem cell-enriched and depleted fractions in cell culture, 100,000 cells from the unfractionated cell, stem cell-enriched and stem cell-depleted fractions were seeded on 6-cm plates with lethally irradiated J2 3T3 feeder cells and cultured in Reinwald–Green medium consisting of F12/DMEM (3:1) supplemented with 5% fetal calf serum, 0.4 µg ml^−1^ hydrocortisone, 8.4 ng ml^−1^ cholera toxin, 5 µg ml^−1^ insulin, 24 µg ml^−1^ adenine and 10 ng ml^−1^ EGF until the colonies expanded. Then, keratinocytes from each fraction were collected for DNA methylation analysis.

### Irradiation of cells.

For X-irradiation, cells were exposed to X-rays in an AGO X-ray System (CP/1601) with 250 kV, 13 mA and 60 cm from the source, giving a dose rate of 0.5 Gy min^−1^. Cells were chronically irradiated by exposure to a gamma-emitting Cs-137 source in a custom-built irradiator (Gemini Technologies) at 37 °C, 5% CO_2_, high humidity and under constant exposure to low levels of ionizing radiation, except when removed for routine media change. Unirradiated controls were cultured in identical culture conditions without ionizing radiation.

### Immunofluorescence.

hESCs and differentiated endothelial cells were seeded on respectively Geltrex- or fibronectin-coated coverslips in appropriate media. When cells were ready for imaging, they were rinsed twice with HBSS with added calcium and magnesium to preserve cell junctions (ThermoFisher, catalog no. 14025050) and fixed in formalin for 10 min. Then cells were permeabilized with 0.1% Triton X-100 in HBSS for 10 min followed by blocking in 2% fetal calf serum in HBSS three times for 10 min each. Coverslips were then incubated with primary antibodies for VE-Cadherin (Santa Cruz Biotechnology, catalog no. sc-9989, dilution 1:100) and OCT4 (Cell Signaling Technologies, catalog no. 2840, dilution 1:100) for 1 h at room temperature. Next, coverslips were subjected to three 10-min washes with 2% fetal calf serum in HBSS and incubated with Alexa-conjugated secondary antibodies for 1 h at room temperature. Then, coverslips were washed again three times for 10 min each in FCS/HBSS and mounted on slide with Vectashield with DAPI (Vector Laboratories, catalog no. H-1500). Imaging was performed by using Nikon Eclipse Ti inverted microscope, and data analysis was performed with NIS-Elements AR Microscope Imaging Software (Nikon).

JC-1 mitochondrial potential sensor (Invitrogen) was reconstituted according to the manufacturer’s instructions. Control or CCCP-treated human keratinocytes were incubated with 1 µM JC-1 for 30 min at 37 °C.

### Determination of cellular mitochondrial function.

Primary cell culture of foreskin keratinocytes was plated in XFp Cell Culture Miniplates (Agilent 103022–100,) coated with collagen/fibronectin matrix (Sigma-Aldrich, C7521 and F0895) at a density of 3 × 10^4^ cells per well. Three wells were used as controls, and three were treated with CCCP (ThermoFisher, 10175140). The cells were treated overnight with 1 μM CCCP in CnT Media (CELLnTEC, CnT-07). Seahorse XF Cell Mito Stress Test Kit (Agilent, 103010–100) was used to measure basal, uncoupled and nonmitochondrial OCRs (pmol min^−1^), proton leak and an estimate of the anaerobic glycolysis based on the H+ protons that are produced for each lactate anion (extracellular acidification rate [ECAR (mpH min^−1^)]) according to manufacturer instructions. Nonmitochondrial respiration was subtracted from all the measurements. Proton leak was calculated as (minimum rate measurements after oligomycin injection) − (nonmitochondrial OCR). ATP production: (basal OCR) − (minimum rate measurements after oligomycin injection). ‘Mito ATP production rate’ and ‘glycol ATP production rate’ are basal measurements of OCR and ECAR.

### Western blotting.

Frozen cell pellets were lysed in 1% SDS, 50 mM Tris, pH 8.0 buffer with added Halt Protease and Phosphatase Inhibitors (ThermoFisher, 10137963), and proteins were quantified by Pierce BCA Protein Assay Kit (ThermoFisher, 23227) according to the manufacturer’s protocol. Protein lysates were separated on 4–20% Mini-PROTEAN TGX Gels (Bio-Rad, 456–1096) and transferred onto polyvinylidene difluoride membrane (Bio-Rad, 170–4156) using semidry Trans-Blot Turbo Transfer System (Bio-Rad) and a high-molecular-weight standard protocol. Membranes were blocked in 5% TBS-T milk at room temperature for a minimum of 1 h with gentle rocking. Blots were incubated with primary antibodies at room temperature overnight with gentle rocking. Blots were washed three times with TBS-T and incubated with secondary antibodies for 1 h at room temperature. The antibodies used are listed in Table 3. After washing three times in TBS-T, blots were imaged using Immobilon Western Chemiluminescent HRP Substrate (Millipore, WBKLS0500).

### Generation and use of recombinant retroviruses.

Retroviruses (carrying the hTERT gene and those carrying activated ras gene) were generated using the Phoenix A packaging cell line (kind gift from the Nolan laboratory). The day before transfection, 4 million Phoenix A cells were seeded in a T-75 flask (Sarstedt, 83.3911.002) in DMEM (Sigma-Aldrich, D6429) supplemented with fetal calf serum (as above) up to 10%. The next day, 10 µg pBabePurohTERT (Addgene) or pBabePuroRasV16 (kind gift from R. Weinberg) were mixed with calcium phosphate reagents, which were procured from Promega (Profection Mammalian Transfection System, Promega, E1200) according to the accompanying instructions. The next day, the media of the cells were removed, the monolayer of cells rinsed once with HBSS (as above) and replaced with media of the target cells (as above). The next day the virus-containing media were collected and filtered through a 0.2-µm filter. Polybrene (Millipore, TR-1003-G) was added up to 8 µg ml^−1^, and the virus-polybrene mixture was added to the target cells. The next day, the media were replaced with fresh media containing 1 µg ml^−1^ puromycin (Invitrogen, A11138–03). Antibiotic selection continued until all uninfected target cells, which were also similarly processed, were dead.

### DNA methylation age analyses.

The primary epigenetic clock used in this work is the Skin&blood clock. The other clocks, namely the Horvath clock, Hannum clock and PhenoAge clocks, were used as comparison, and the results from these clocks were not used to formulate any conclusions. The R software code of the Skin&blood clock can be found in the supplement of the original paper^16^. Similarly, the R software scripts underlying the pan tissue clock^1^, the PhenoAge clock^40^ and the DNA methylation estimate of the telomere length^9^ can be found in the supplements of the respective citations. Online software for calculating these biomarkers can be found at https://DNAmAge.genetics.ucla.edu/new. Finally, a GitHub page created by another group distributes software code for these clocks (https://github.com/isglobal-brge/methylclock).

### Statistics and reproducibility.

No statistical method was used to predetermine sample size due to the nature of the experiments. In particular, cells isolated from different individuals exhibit notable biological differences (e.g., in terms of proliferation capacity and rate of proliferation). This difference is also manifested in the way the cells respond to interventions and tests. This difference, however, is only in terms of magnitude of change (or response) and not in terms of direction of change. Therefore, whereas the results presented here are all reproducible between cells from different donors, the extent of their responses to testing can be quite broad. This is the unavoidable nature of experimentation with primary cells, as opposed to cell lines, which have undergone extensive selection. All experiments were carried out multiple times, although only a single representative of these experiments is shown. The number of repeats (always with cells from different donors) is provided in the figure legends. Following similar results from more than at least two repeats (most often more than three) with cells from different donors, the observation was accepted to be valid and correct. No data were excluded from the analyses, the experiments were not randomized and the investigators were not blinded to allocation during experiments and outcome assessment.

### Reporting Summary.

Further information on research design is available in the Nature Research Reporting Summary linked to this article.

## Data availability

DNA methylation profiles have been submitted in a public repository (Gene Expression Omnibus). The data can be accessed via Gene Expression Omnibus submissions GSE197719 and GSE197723 or SuperSeries GSE197724. Other data underlying the study are available as Source Data files or from the corresponding author upon reasonable request.

## Extended Data

**Extended Data Fig. 1 |:**
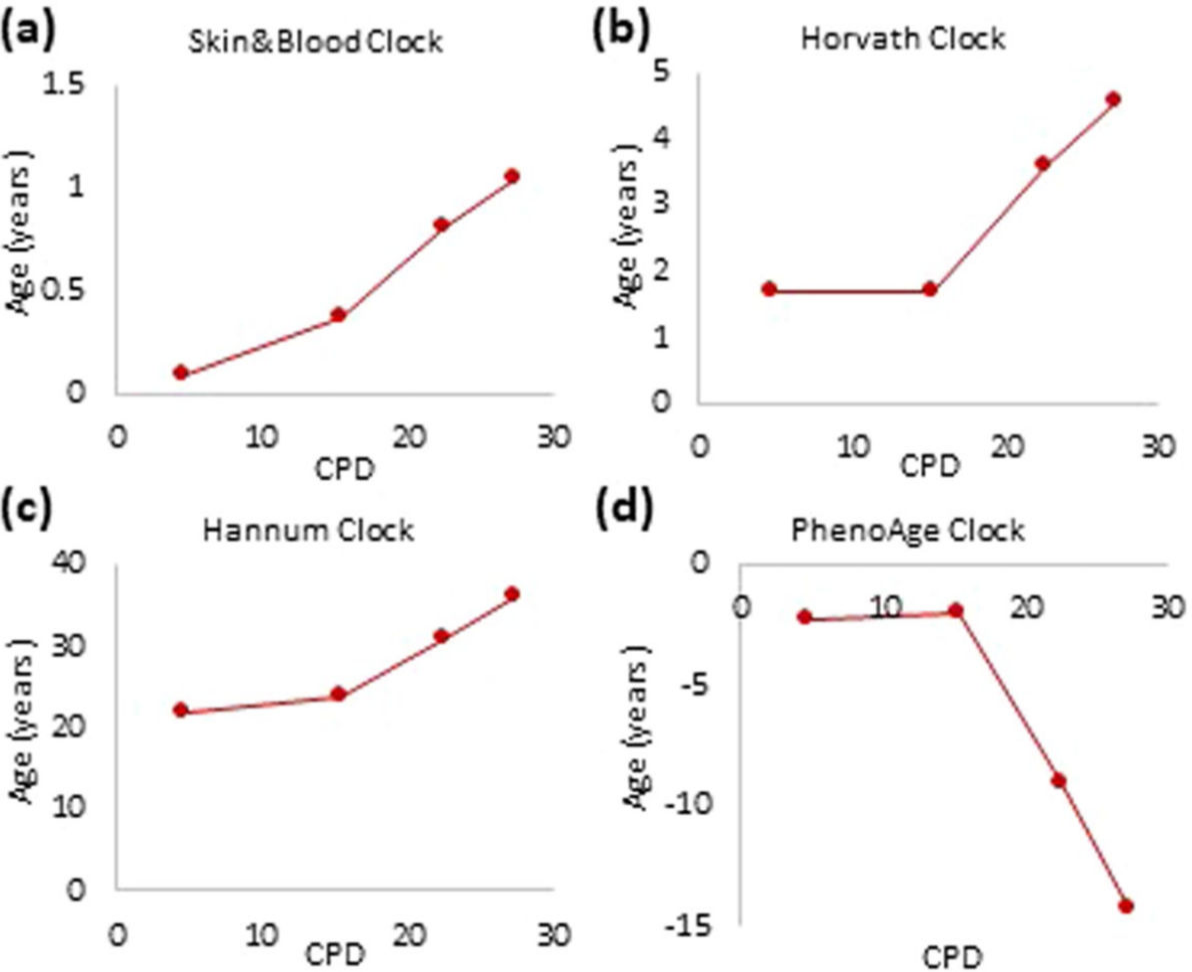
Comparison between the performances of four most-commonly employed epigenetic clocks on *in vitro* cultured human cells. Measurement of EpiAge of primary keratinocytes from human neonatal foreskin that were cultured *in vitro* and monitored at different points in time. DNA methylation profiles of these cells were analyzed using the **(a)** Skin&blood clock, **(b)** Horvath clock, **(c)** Hannum clock and **(d)** PhenoAge clock. Extended_Data_Figure_Legend.docx.

**Extended Data Fig. 2 |:**
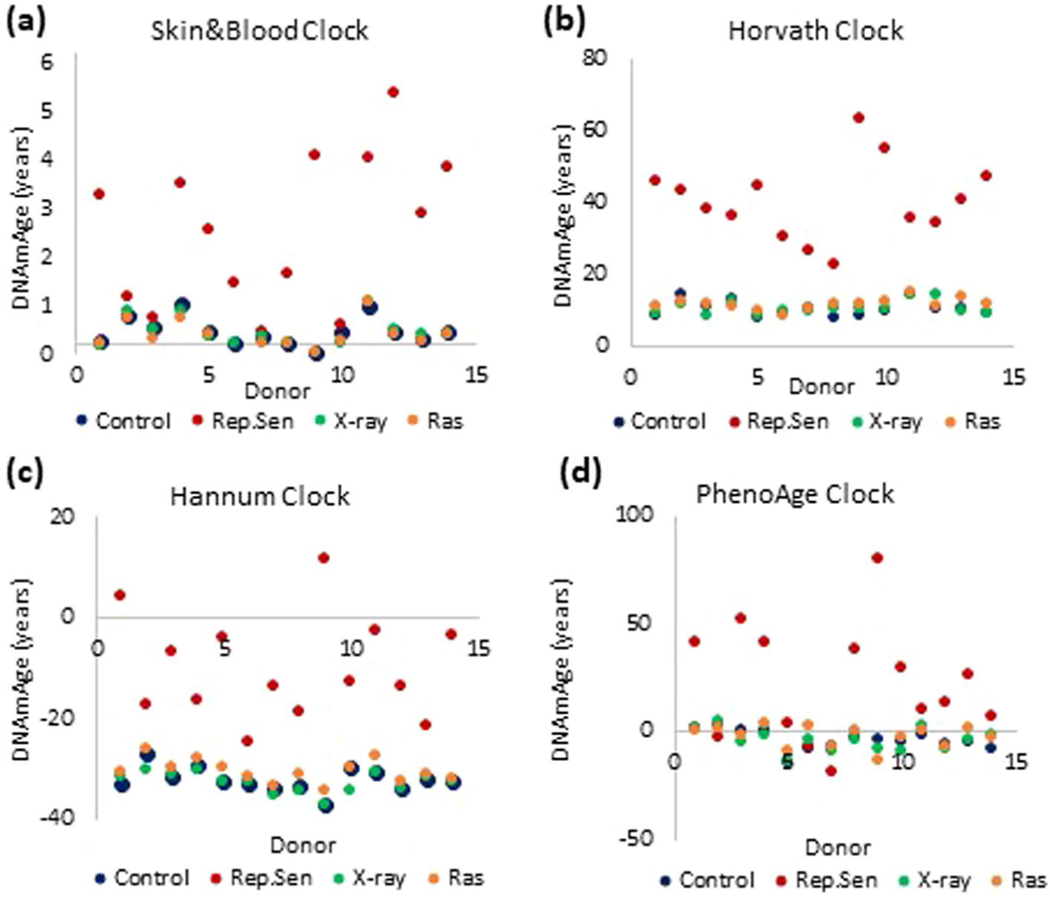
effects of various inducers of cellular senescence on epigenetic aging. Measurement of EpiAge with four epigenetic clocks on primary human fibroblasts isolated from neonatal foreskins of 14 donors. Fibroblasts were cultured until replicative senescence (red), induced to senesce by X-irradiation (green), induced to senesce by ectopic expression of activated ras oncogene (orange) or untreated (blue). Methylation profiles of these cells were analyzed using the **(a)** Skin&blood clock, **(b)** Horvath clock, **(c)** Hannum clock and **(d)** PhenoAge clock.

**Extended Data Fig. 3 |:**
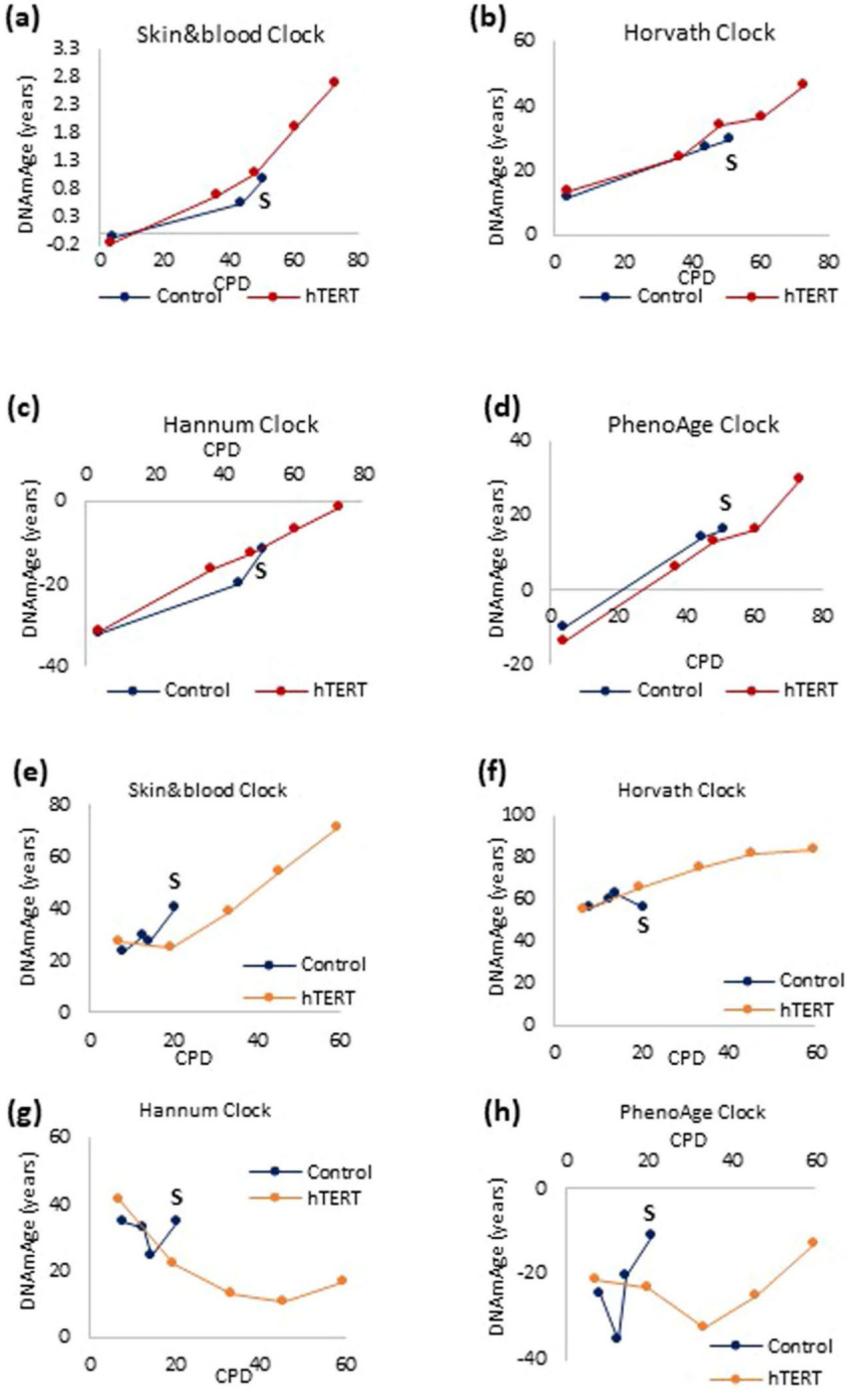
The impact of prevention of replicative senescence on epigenetic aging. Measurement of EpiAge of human neonatal primary fibroblasts transduced with hTERT-expressing vectors or empty vector at various time point during their culture until senescence (S) of the control cells. DNA methylation profiles were analyzed by four different epigenetic clocks **(a-d)**. Similar approach was employed with adult human coronary artery cells **(e-h)**.

**Extended Data Fig. 4 |:**
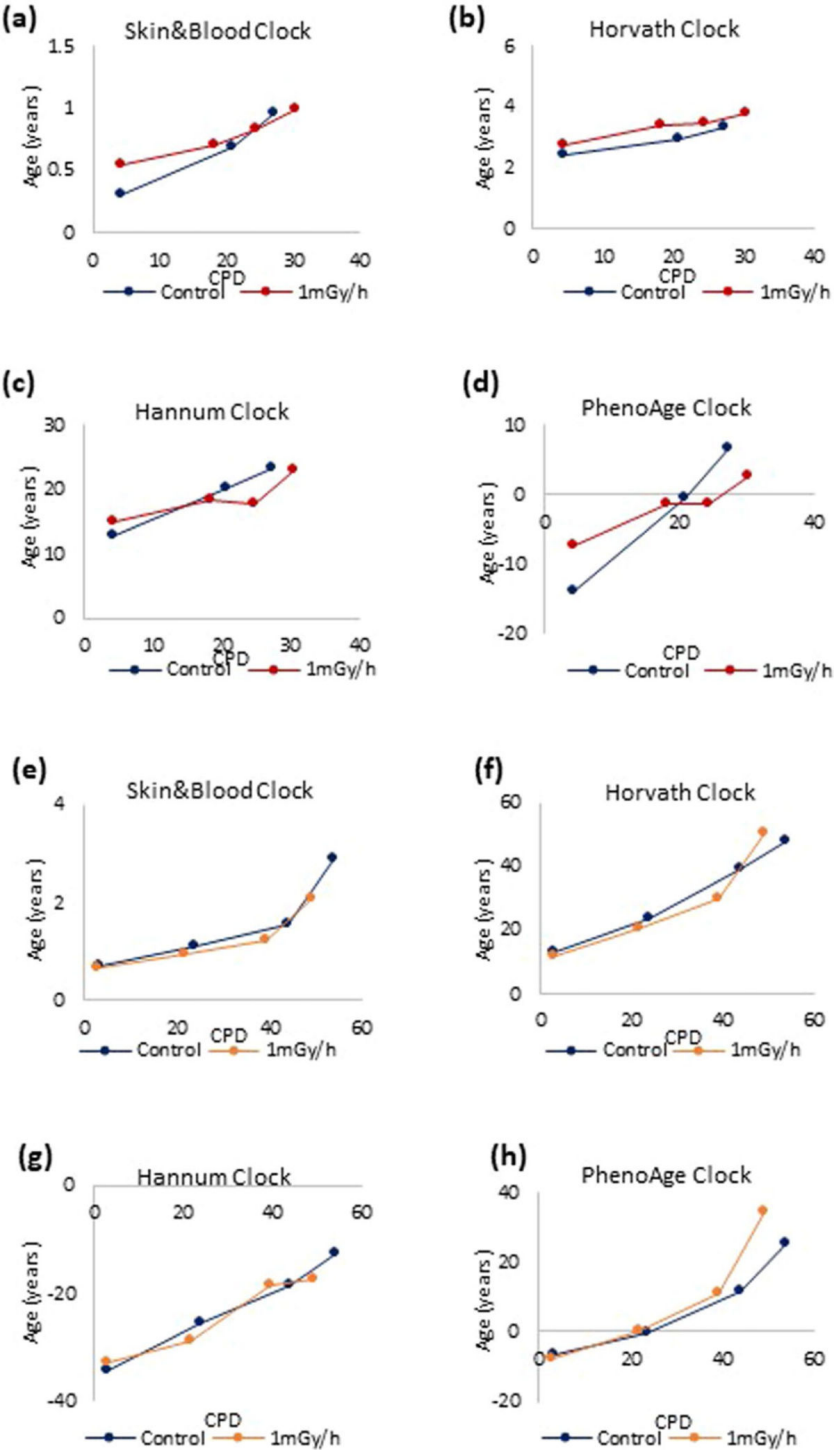
The impact of chronic ionizing radiation on epigenetic aging. Primary human keratinocytes **(a-d)** and primary human fibroblasts **(e-h)** were subjected continuous gamma irradiation at 1 mGy/hr until replicative senescence. DNA methylation profiles of these cells and their respective unexposed controls that were cultured in parallel were analyzed by four different epigenetic clocks.

**Extended Data Fig. 5 |:**
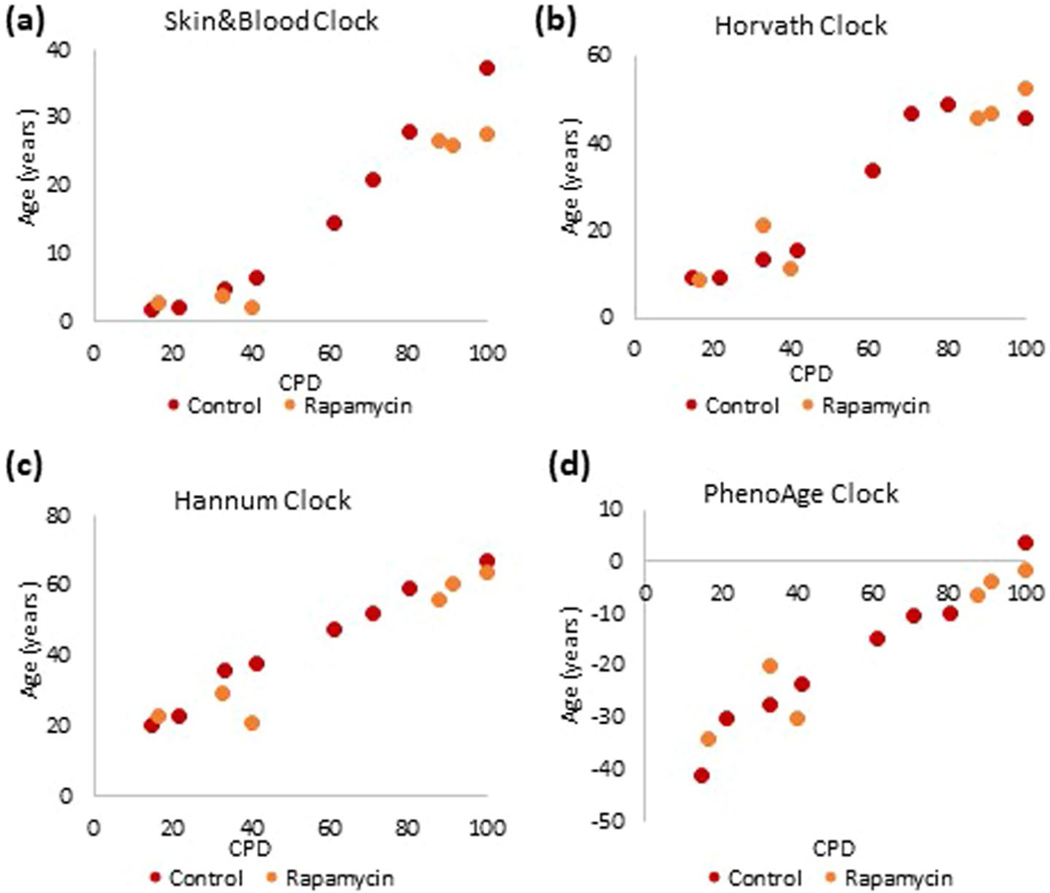
The impact of perturbing nutrient-sensing mechanism at different cellular age. DNA methylation profiles of human umbilical vein endothelial cells cultured with rapamycin at early (E) or late (L) time points of culture (corresponding to young and old cells respectively) were subjected to EpiAge measurement using four different epigenetic clocks **(a-d)**.

**Extended Data Fig. 6 |:**
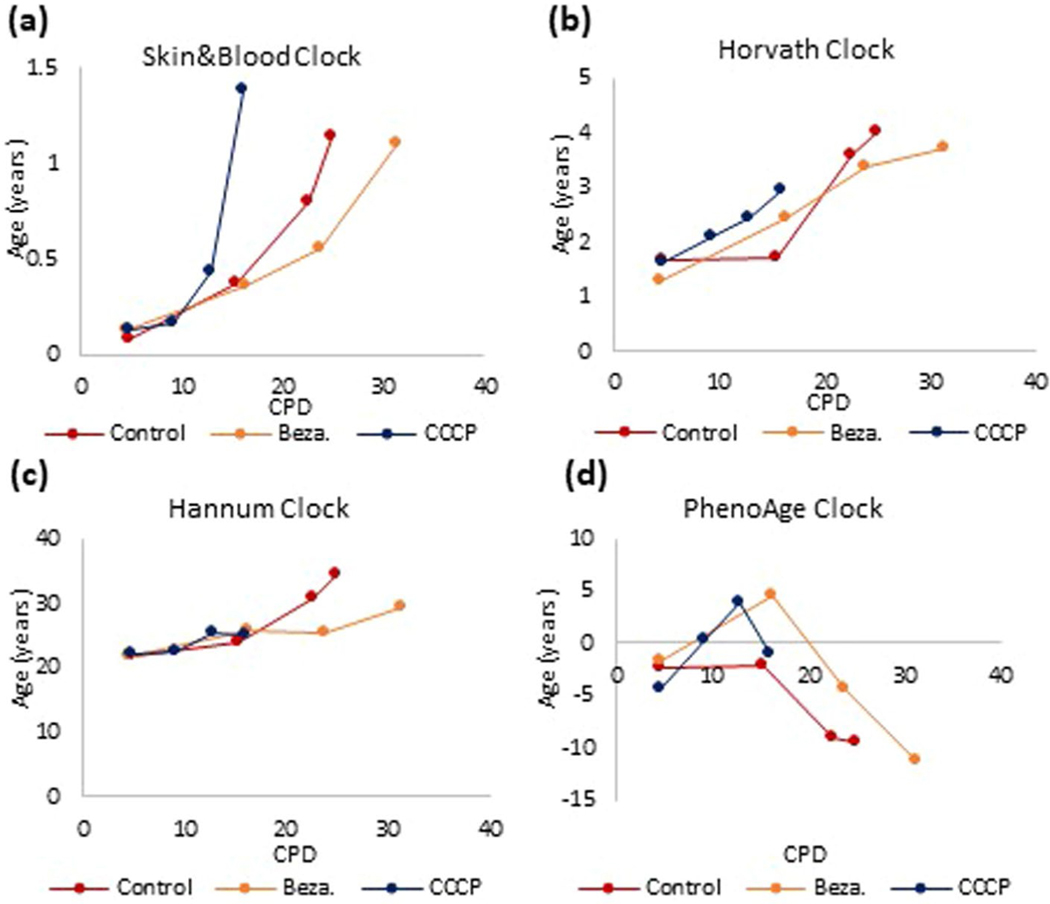
The impact of perturbing mitochondria function on epigenetic aging. Mitochondrial activity of primary human keratinocytes was either compromised by treatment with CCCP or enhanced by culturing in Bezafibrate. DNA methylation profiles of these cells and their untreated controls were analyzed by four different epigenetic clocks **(a-d)**.

**Extended Data Fig. 7 |:**
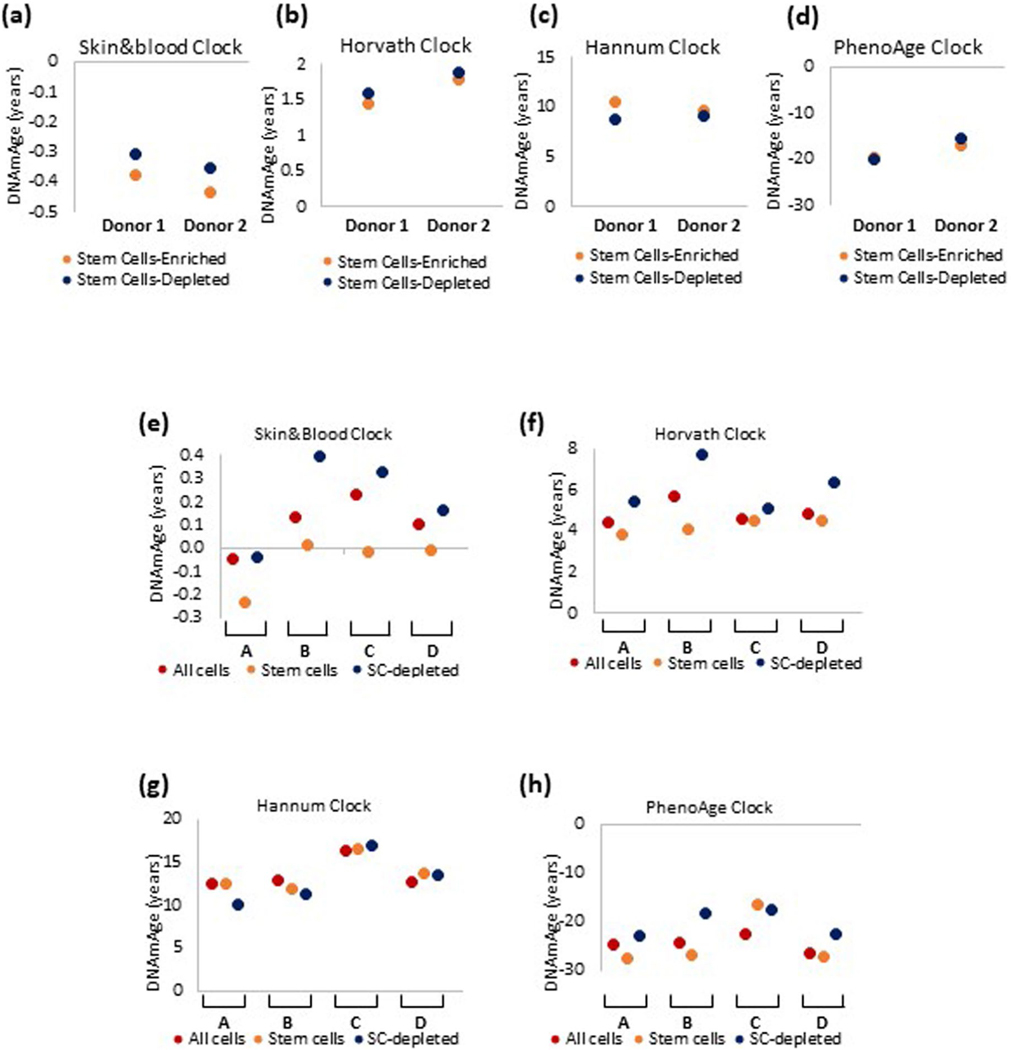
epigenetic age of epidermal stem cells and non-stem cells. Epigenetic age of stem cell-enriched and stem cell-depleted keratinocytes isolated from two different (Donor 1 and 2) neonatal foreskins, and measured by four epigenetic clocks **(a-d)**. Epigenetic age of stem cell-enriched and stem cell-depleted keratinocytes isolated from foreskins of four neonate donors (A-D), that were put to proliferate in culture. Epigenetic age was measured using four different epigenetic clocks **(e-h)**.

**Extended Data Fig. 8 |:**
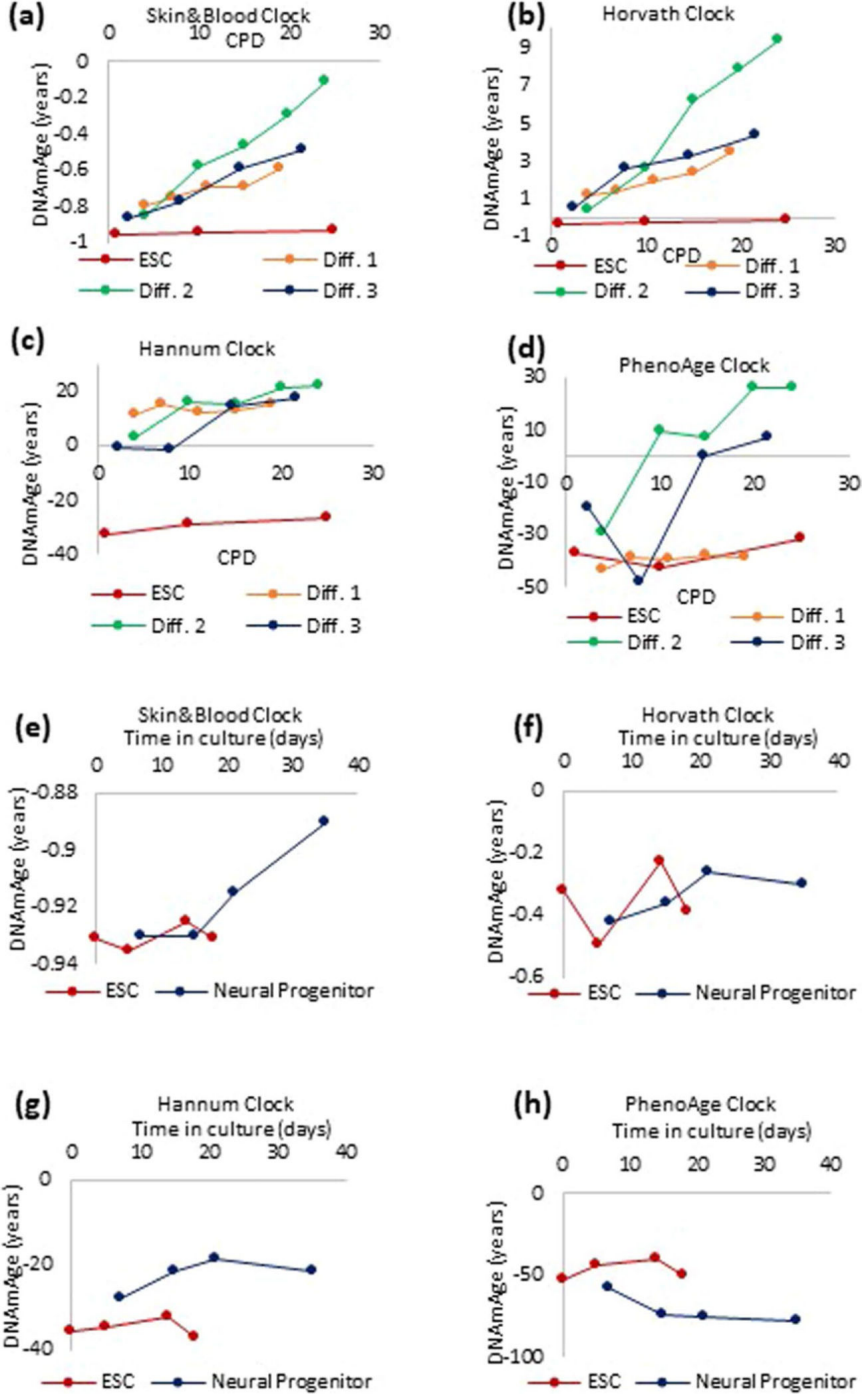
initiation of the ticking of the epigenetic clock. Human embryonic stem cells (ESCs) were differentiated *in vitro*, into endothelial cells (Diff 1–3 are three independent EC lines derived) **(a-d)**, or neural progenitor cells **(e-h)**, and the EpiAge of these cells were measured using four different epigenetic clocks.

**Extended Data Fig. 9 |:**
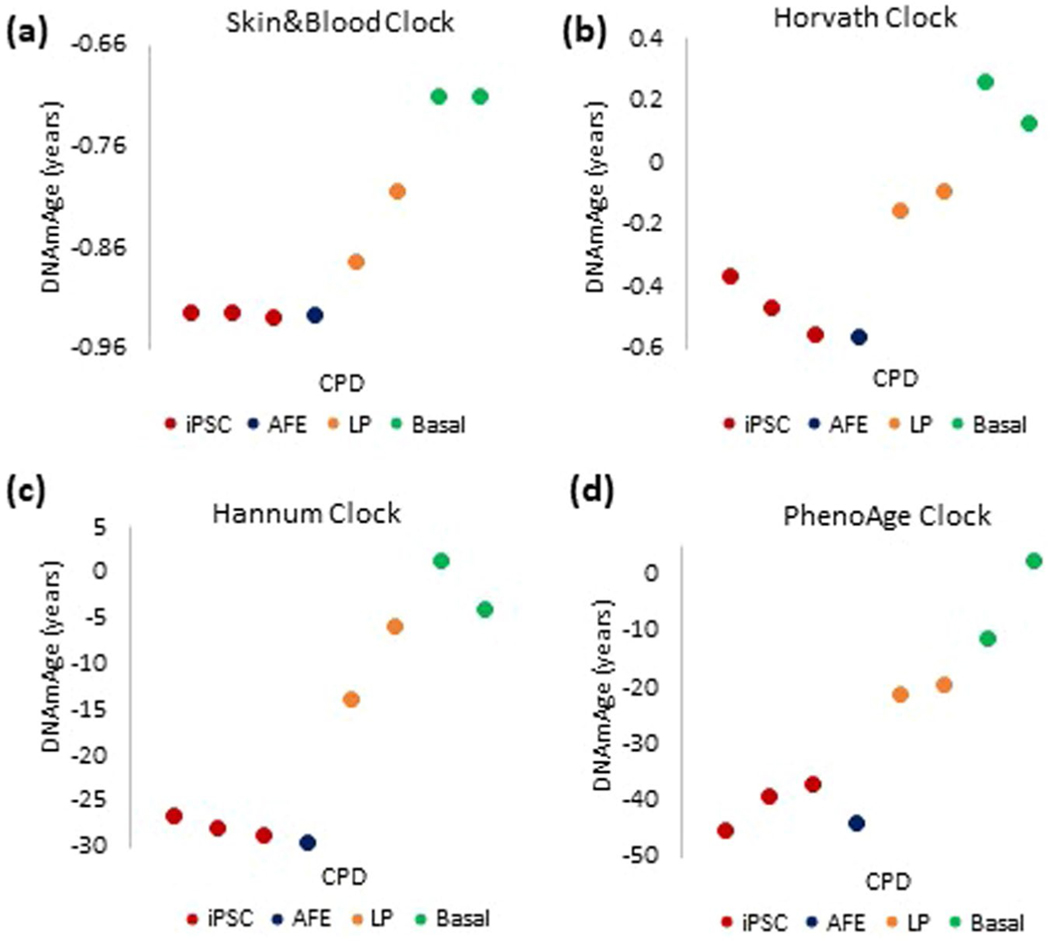
initiation of the epigenetic clock during iPSC differentiation. Tracking of EpiAge of human induced pluripotent stem cells (iPSC) that were differentiated into human lung epithelial basal cells (Basal) via transition through anterior foregut cells (AFE) and lung progenitor cells (LP). The EpiAge of these cells were measured using the four different epigenetic clocks **(a-d)**.

**Extended Data Fig. 10 |:**
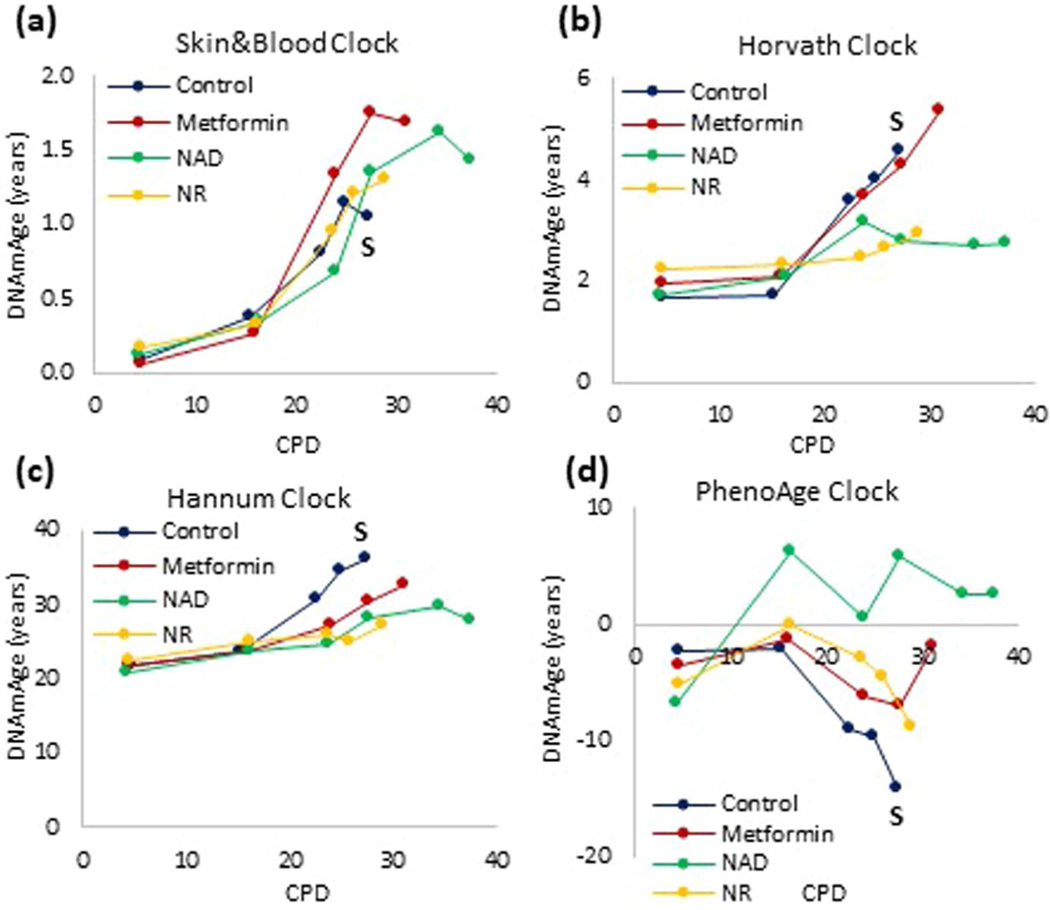
Distinct effects of various compound on lifespan and aging. Epigenetic age of human neonatal keratinocytes treated continuously with nicotinamide adenine diphosphate (NAD), nicotinamide riboside (NR) or metformin. The arrow and letter ‘S’ denotes when untreated control cells became senescent. The DNA methylation profiles of these cells were analyzed by four different epigenetic clocks **(a-d)**.

## Figures and Tables

**Fig. 1 |:**
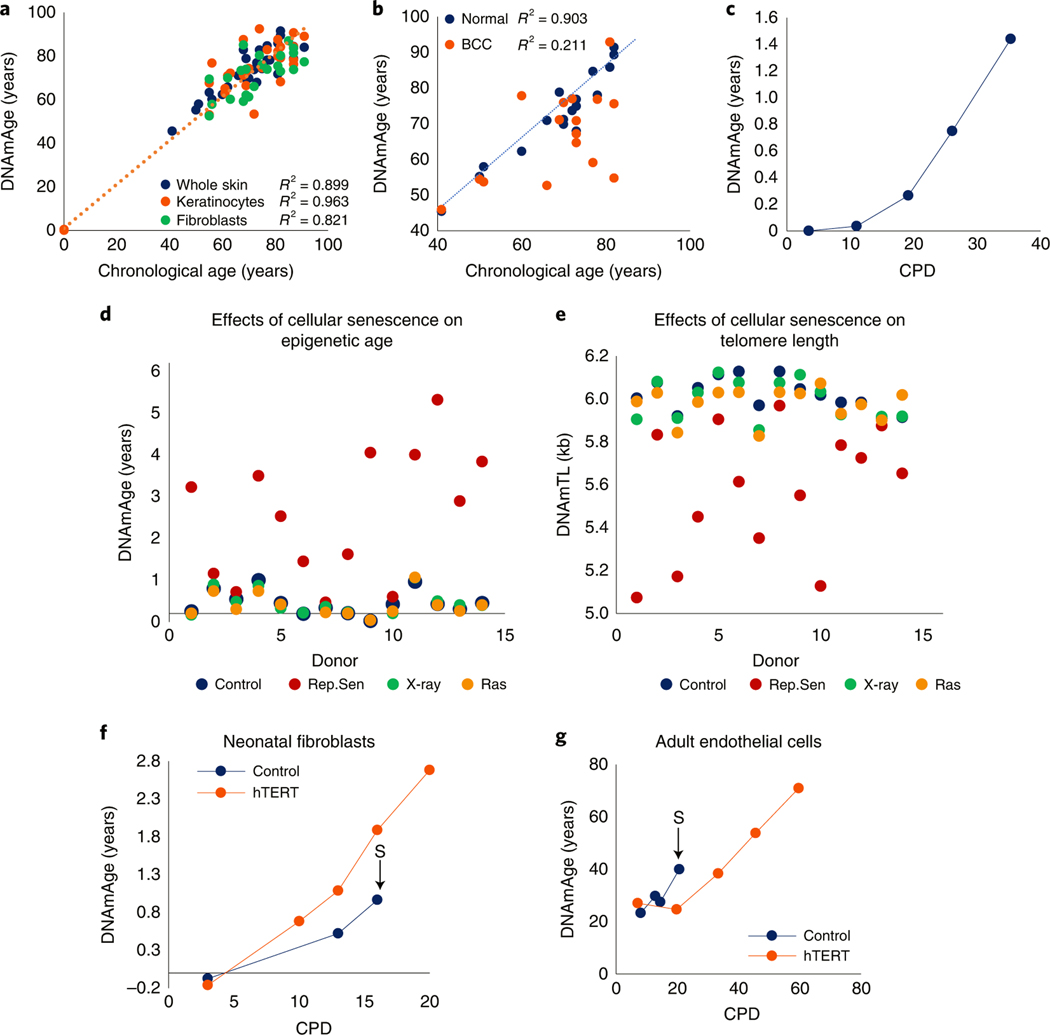
EpiAge is distinct from cellular senescence and telomere attrition. **a**, Measurement of EpiAge (DNAmAge) of whole skin, keratinocytes and fibroblasts from 14 healthy human skin samples. Absence of samples between 1 and 40 years old reflects the scarcity of hospital admissions of this demographic group. **b**, Measurement of DNAmAge of 17 BCCs and corresponding adjacent healthy skin. **c**, EpiAge of in vitro-cultured neonatal human dermal keratinocytes (HDK) derived from foreskin. CPD, cumulative population doubling. Representative of more than three experiments. **d**, EpiAges of primary human dermal fibroblasts isolated from skin of 14 healthy neonatal donors and subjected to 20 Gy X-rays, transduced to express oncogenic ras or cultured until replicative senescence (Rep.Sen). **e**, DNA methylation-based estimation of telomere length (DNAmTL) of cells described in **d. f**, DNAmAge of neonatal primary human dermal fibroblasts transduced with empty vector (control) or hTERT-expressing vector (hTERT). The arrow and letter ‘S’ denote the point at which the untreated control cells became senescent. Representative of two experiments. **g**, EpiAge of adult HCAECs transduced with empty vector (control) or vector expressing hTERT. Representative of three experiments

**Fig. 2 |:**
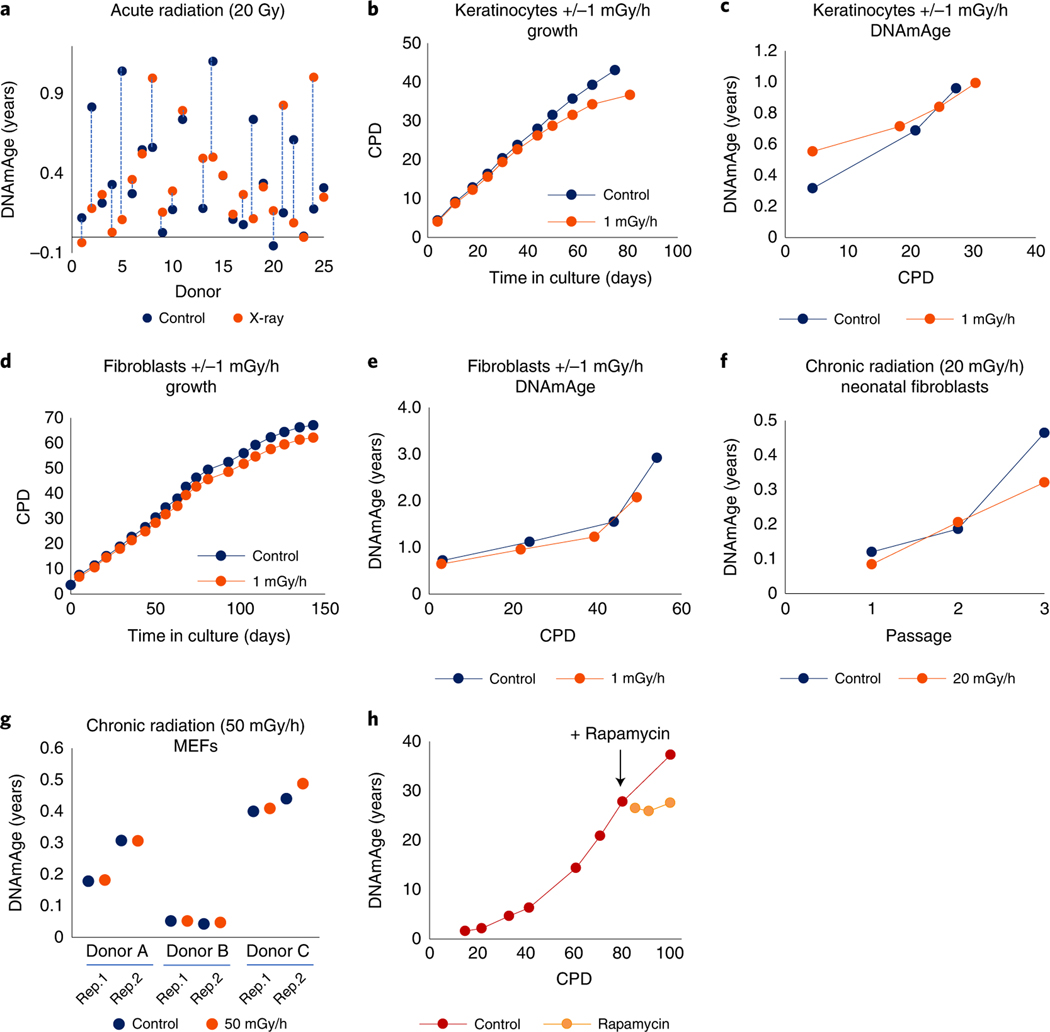
EpiAge is not affected by genomic instability induced by radiation-induced DNA breaks. **a**, Measurement of EpiAge of neonatal HDFs from 25 donors, with ages of unirradiated cells (control) and their corresponding 20 Gy-irradiated (X-ray) counterparts connected by vertical lines. **b**, Effects of continuous low-dose radiation (1 mGy/h) on the growth of neonatal HDKs. **c**, Effects of continuous radiation (1 mGy/h) on EpiAge of neonatal HDK described in **b**. **d**, Effects of continuous low-dose radiation (1 mGy/h) on the growth of neonatal HDFs. **e**, Effects of continuous radiation (1 mGy/h) on EpiAge of neonatal HDFs as described in **d**. For **b**–**e**, figures are representative of three experiments carried out at the same time with cells from different donors. **f**, Effects of chronic irradiation at 20 mGy/h on EpiAge of neonatal HDFs through three passages in culture in a single experiment. **g**, Measurement of EpiAge of three independent strains of MEFs with replications 1 and 2 (Rep.1 and Rep.2). Cells were either unirradiated (control) or irradiated at (50 mGy/h) for 24 h followed by 6 days of recovery without irradiation. Representative of a single experiment. **h**, Effects of rapamycin on EpiAge of hTERT-immortalized HUVECs at adult EpiAge. Rapamycin was administered at the point indicated by arrow (+ Rapamycin). Representative of two experiments, with the other experiment using keratinocytes.

**Fig. 3 |:**
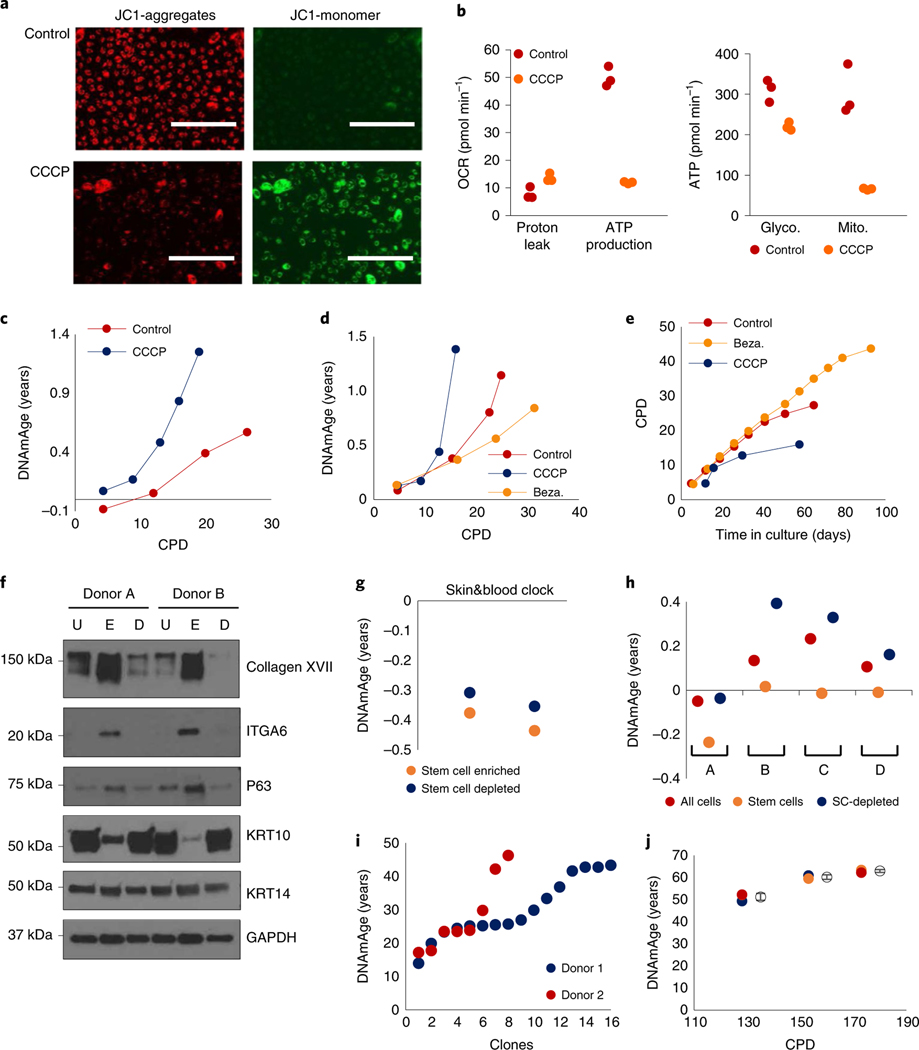
Mitochondrial activity and tissue composition affects EpiAge. **a**, Mitochondrial potential of untreated and CCCP-treated neonatal HDKs compared using JC-1, which aggregates in mitochondria with high potential (red) but remains green when unaggregated Scale bars, 200 μm. Representative of two experiments. **b**, Production of adenosine triphosphate (ATP) and mitochondrial proton leak of control and CCCP-treated HDKs were measured with Seahorse technology (left). Comparative ATP production from glycolysis or the Krebs cycle was also measured (right). Data represent triplicates of a single experiment, which was repeated twice. OCR, oxygen consumption rate. **c**, Determination of the effect of CCCP on cellular epigenetic aging. Representative of a single experiment. **d**, Effects of CCCP and bezafibrate (Beza.) on epigenetic aging of neonatal HDK (representative of two experiments). **e**, Lifespan of HDKs (representative of a single experiment). Representative figure of two experiments. **f**, Expression of various proteins in unprocessed epidermis (U), stem cell-enriched fraction (E) and stem cell-depleted fraction (D) from two neonatal donors. Representative of four blots. **g**, EpiAge of stem cell-enriched fraction (stem cells) and stem cell-depleted fraction (SC-depleted) in epidermis from two donors. Representative of a single experiment. **h**, EpiAges of in vitro-cultured unfractionated cells (all cells), stem cell-enriched fraction (stem cells) and stem cell-depleted fraction (SC-depleted) from four neonatal donors, A to D. Representative of a single experiment. **i**, EpiAge of individual clones derived from two independent HCAEC donors with an EpiAge of 23 years. **j**, EpiAge of HUVECs from three different CPDs was measured in triplicate. Empty circles represent mean EpiAge of the replicates, and error bars represent standard deviation. Representative of a single experiment

**Fig. 4 |:**
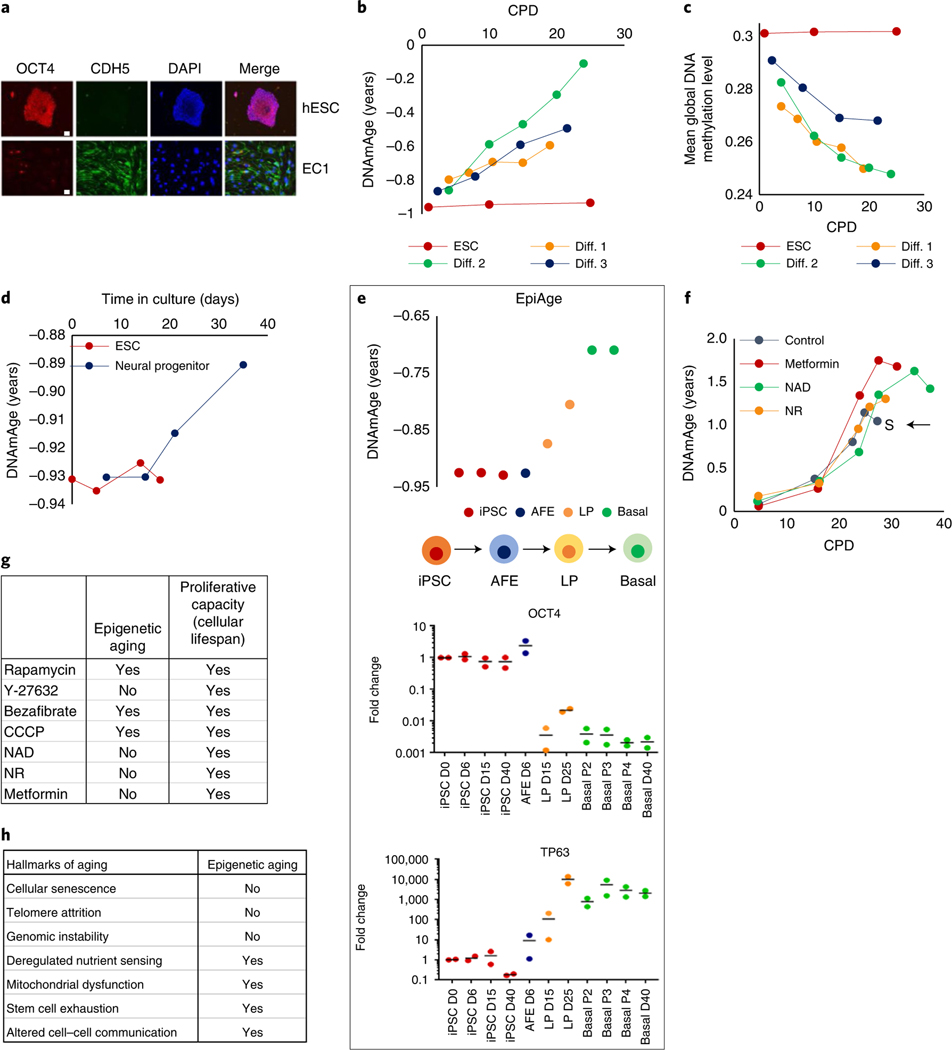
Epigenetic clock starts ticking when ESCs lose their pluripotency. **a**, Immunofluorescence analyses of OCT4 (red), expressed exclusively by human ESCs (hESCs) and VE-cadherin (CDH5) in green, a marker of endothelial cells. EC1 cells were differentiated from hESCs to become endothelial cells. Red dots in EC1 are anti-CD31 beads used in endothelial cell purification, which were stained by secondary antibody. Representative of two experiments. Scale bars, 20 μm. **b**, EpiAge of three independent lines of endothelial cells (Diff.1–3) differentiated from hESCs. **c**, Mean methylation levels of the cells described in **b**. **d**, EpiAge following differentiation of hESCs into neural progenitor cells. Representative of a single experiment. **e**, Tracking of EpiAge of human iPSCs that were differentiated into human lung epithelial basal cells (basal) via transition through AFE and LP cells. RnA levels of OCT4 and TP63 of these cells were measured by qPCR. Representative of two experiments. D, day; P, passage. **f**, EpiAge of human neonatal keratinocytes treated continuously with nAD, nR or metformin. The arrow and letter ‘S’ denote when untreated control cells became senescent. Representative of two experiments. **g**, Comparison of the effects of tested compounds on epigenetic aging and cellular population lifespan. **h**, Summary of the relationship between the hallmarks of aging and epigenetic aging.

**Table 1 |:** growth factors used for AFe differentiation.

Growth factor	Manufacturer, catalog no.	Days 0–1	Days 1–3	Days 3–4	Days 4–6

Wnt3a	R&D Systems, 5036-GMP	50 ng ml^−1^	-	-	-
Activin A	R&D Systems, 338-AC	100 ng ml^−1^	100 ng ml^−1^	-	-
Dorsomorphin	Torcis, 3093	-	-	1μM	-
SB431542	Torcis, 1614	-	-	5μM	5μM
IWP2	Torcis, 3533	-	-	-	1μM

**Table 2 |:** Primers used in gene expression experiment.

Primer name	Primer sequence (5′−3′)

OCT4-F	GACAGGGGGAGGGGAGGAGCTAGG
OCT4-R	CTTCCCTCCAACCAGTTGCCCCAAAC
P63-F	CCACCTG GACGTATTCCACTG
P63-R	TCGAATCAAATGACTAGGAGGGG
GADPH-F	GACATCAAGAAGGTGGTGAA
GADPH-R	TGTCATACCAGGAAATGAGC

**Table 3 |:** Primary antibodies used in western blotting.

Antibody	Dilution	Species	Protein lysate	Manufacturer (catalog no.)

Collagen XVII	1:5,000	Rabbit	10 μg	Abcam (ab184996)
Integrin 6α	1:10,000	Rabbit	10 μg	Abcam (ab181551)
P63	1:5,000	Mouse	10 μg	Santa Cruz Biotechnology (sc-8431)
Cytokeratin 14	1:10,000	Mouse	2 μg	Abcam (ab7800)
Keratin 10	1:5,000	Mouse	2 μg	Abcam (ab76318)
GAPDH	1:50,000	Mouse	2 and 10 μg	Proteintech (60004–1-Ig)
